# A proposal of *Bursaphelenchus uncispicularis* Zhuo, Li, Li, Yu & Liao, 2007 as a junior synonyms of *B. yongensis* Gu, Braasch, Burgermeister, Brandstetter & Zhang, 2006

**DOI:** 10.21307/jofnem-2020-130

**Published:** 2021-01-13

**Authors:** Jianfeng Gu, Kan Zhuo, Jinling Liao

**Affiliations:** 1 Ningbo Customs Technology Center (Ningbo Inspection and Quarantine Science Technology Academy), Huikang Road 8, Ningbo 315100, Zhejiang, P. R. China; 2 Laboratory of Plant Nematology, South China Agricultural University, Guangzhou, 510642, Guangdong, P. R. China

**Keywords:** China, Papillae, Taxonomy

## Abstract

*Bursaphelenchus yongensis* was first reported in China, and later found in Japan and Korea. It is characterized by a relatively slim body (a = 42 and 57 for females and males, respectively). The excretory pore is located at level of median bulb, the lateral field has three lines, and a small vulval flap is present. A long post-uterine branch extends 2/3 to 3/4 of the vulva to anus distance. The conoid female tail has a 2–5 µm long mucron in the central position at the terminus. Spicules are small, condylus high and strongly dorsally bent. Subsequently *Bursaphelenchus uncispicularis* was described from China. Both morphological characters and morphometrics are very similar to *B. yongensis*, except for the number of lateral lines (4 vs 3) and male caudal papillae (7 vs 4). Re-examination of type material and a Beijing population of *B. yongensis* determined that *B. yongensis* has 7 caudal papillae instead of 4 as originally reported. It is possible that the poor condition of the type specimens of *B. uncispicularis* could have created difficulty in the determination of lateral line number. Unfortunately, type material of *B. uncispicularis* has been lost. Therefore, there is no evidence that *B. uncispicularis* exists. It is now established that *B. yongensis* is present in China, Japan and Korea with a common host species (*P. thunbergii*) and a common widespread vector (*Cryphalus fulvus*). Therefore, based on the geographic, ecological, molecular, and morphological data, we propose *Bursaphelenchus uncispicularis* Zhuo, Li, Li, Yu & Liao, 2007 as a junior synonym of *B. yongensis* Gu, Braasch, Burgermeister, Brandstetter & Zhang, 2006.

*Bursaphelenchus yongensis* Gu, Braasch, Burgermeister, Brandstetter & Zhang, 2006 (Gu et al., 2006) was originally described from *Pinus massoniana* Lamb. in Ningbo city, Zhejiang province, China. It is characterized by a relatively slim body (a = 42 and 57 for females and males, respectively), excretory pore located at level of median bulb, lateral field with three lines, small vulval flap present, long post-uterine branch extending 2/3 to 3/4 of the vulva to anus distance and a conoid female tail showing a 2–5 µm long mucron in central position at the terminus. SEM pictures and ITS-RFLP pattern were provided in this paper. Later, the sequences of the partial 18 S, ITS1/2 and 28 S D2-D3 region of *B. yongensis* were deposited in Genbank with accession numbers AM397023, AM180513 and AM396581, respectively.

About half a year following the report of *B. yongensis*, a new *Bursaphelenchus* species named *B. uncispicularis* Zhuo, Li, Li, Yu & Liao, 2007 was described, which was isolated from *P. yunnanensis* Franch. in Longling county, Yunnan province, China. Both morphological characters and morphometrics are very similar to *B. yongensis*, except the number of lateral lines (4 vs 3) and male caudal papillae (7 vs 4). Since both original descriptions were under review at the same time, neither was able to include a comparison between *B. uncispicularis* and *B. yongensis*. The original specimens of *B. uncispicularis* were not in good condition. Zhuo et al. (2007) provided only the drawing and the male tail picture under light microscope, lacking SEM images and molecular data. Unfortunately, the type material kept in Plant Nematode Research Laboratory, South China Agricultural University, Guangzhou, China is missing. We have tried to re-isolate *B. uncispicularis* from the type locality but failed.

Kanzaki et al. (2010) found *B. yongensis* from the underside of the elytra of *Cryphalus fulvus* Niijima, which emerged from a dead log of *P. thunbergii* Parl. collected at Higashi-Ichiki, Hioki, Kagoshima Prefecture, Japan. The molecular sequences of 28 S D2/D3 region (AM396581) and ITS1/2 (AM180513) were almost identical to those of the Chinese population. However, Kanzaki et al. (2010) provided several morphological differences in male tail and female reproductive tract. A pair of minute P4 were found at the base of the bursa. Although P1 was not described, it could be seen in Figure 1D (Kanzaki et al., 2010). In females, a three-celled structure, which was not mentioned in the original description, was observed on the dorsal wall of the reproductive tract at the uterus/post-uterine sac junction.

**Figure 1:**
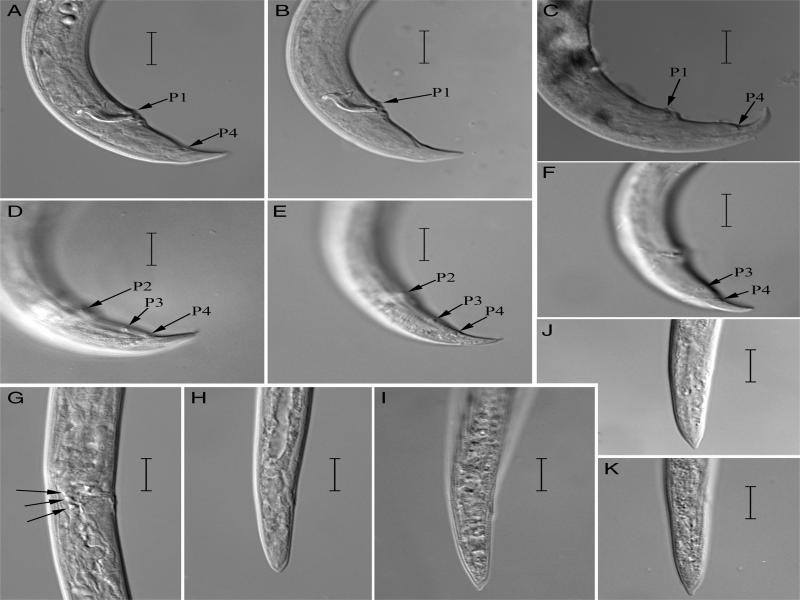
*Bursaphelenchus yongensis* paratypes (C, F) and a population from Beijing (A, B, D, E, G-K): A-F: Male tail by different focus show caudal papillae; G: Lateral view of female vulval regions show a three-celled structure; H-K: Female tail. (Scale bars = 10 μm)

Later, *B. yongensis* was also found from a dead pitch pine (*P. rigida* Mill.) in Daejeon city, South Korea (Han, 2015). The ITS sequence of Korean isolate (KJ857070) showed 99% similarity to that from Chinese isolate in GenBank (AM180513). ITS-RFLP patterns matched 100% with those previously reported for *B. yongensis*. Interestingly, they proved that *B. yongensis* was pathogenic on *Larix leptolepis* Sieb. et Zucc (Han, 2015).

To confirm the male papillae numbers and other characters, paratypes deposited in the Ningbo Customs Technology Center were re-examined. A three-celled structure of the female, which was not mentioned in the original description, was observed. Though very small, but P1 does exist. Minute P4 gland papillae at the base of the bursa were also found, possessing internal connections like secretory duct structures. (Figure 1, arrows show the P1, P2, P3, and P4 papillae).

Recently, *B. yongensis* was also detected from *P. thunbergii* in Beijing, China. The following characters were confirmed again: 3 lateral lines, 7 papillae, a three-celled structure was observed on the dorsal wall of the reproductive tract at the uterus/post-uterine sac junction (Figure 1). Small variation of female tail tip in Beijing population exist, with most females showing a conoid tail with a 2–5 µm long mucron in central position at the terminus. Several showed a bluntly pointed terminus, but never broadly rounded.

According to Kanzaki et al. (2018), both *B. uncispicularis* and *B. yongensis* are now in the *B. eggersi*-group sensu Ryss & Subbotin (2017), subgroup 3, together with *B. carpini* Kanzaki, Masuya, Ichihara, Maehara, Aikawa, Ekino, Taisuke & Ide, 2018 (Kanzaki et al., 2018), *B. clavicauda* Kanzaki, Maehara & Masuya, 2007 (Kanzaki et al., 2007) and *B. cryphali* (Fuchs, 1930) Rühm, 1956, which is characterized by a spicule with a short, wide blade and strongly dorsally recurved condylus with a pointed tip and broad female tail. Except *B. uncispicularis*, all other species share 3 lateral lines, which indicates that the 4 lines of *B. uncispicularis* may have been misjudged. All species in this group have 7 male papillae.

*B. yongensis* is present in China, Japan and Korea with a common host species (*P. thunbergii*) and a common widespread vector (*C. fulvus*). Based on the geographic, ecological, molecular, and morphological data, we propose *Bursaphelenchus uncispicularis* Zhuo, Li, Li, Yu & Liao, 2007 as a junior synonyms of *B. yongensis* Gu, Braasch, Burgermeister, Brandstetter & Zhang, 2006.
